# Supplementary material for: Iterative improvement in the automatic modular design of robot swarms
Source: PeerJ Comput Sci. 2020 Dec 7;6:e322. doi: 10.7717/peerj-cs.322 (PMC7924708; doi:10.7717/peerj-cs.322)
Supplement: Supplemental Information 3 [file peerj-cs-06-322-s003.zip › argos3/doc/api/standalone/a00463_source.html]

ARGoS: plugins/robots/foot-bot/simulator/footbot\_distance\_scanner\_rotzonly\_sensor.h Source File


- Main Page
- Related Pages
- Namespaces
- Classes
- Files

- File List
- File Members

# plugins/robots/foot-bot/simulator/footbot\_distance\_scanner\_rotzonly\_sensor.h

Go to the documentation of this file.

```
00001 
00007 #ifndef FOOTBOT_DISTANCE_SCANNER_SENSOR_H
00008 #define FOOTBOT_DISTANCE_SCANNER_SENSOR_H
00009 
00010 namespace argos {
00011    class CFootBotDistanceScannerRotZOnlySensor;
00012    class CControllableEntity;
00013    class CSpace;
00014 }
00015 
00016 #include <argos3/plugins/robots/foot-bot/control_interface/ci_footbot_distance_scanner_sensor.h>
00017 #include <argos3/plugins/robots/foot-bot/simulator/footbot_distance_scanner_equipped_entity.h>
00018 #include <argos3/core/simulator/entity/embodied_entity.h>
00019 #include <argos3/core/utility/math/rng.h>
00020 #include <argos3/core/utility/math/ray3.h>
00021 #include <argos3/core/simulator/sensor.h>
00022 
00023 #include <string>
00024 #include <map>
00025 
00026 namespace argos {
00027    
00028    class CFootBotDistanceScannerRotZOnlySensor : public CSimulatedSensor,
00029                                                  public CCI_FootBotDistanceScannerSensor {
00030 
00031    public:
00032 
00033       CFootBotDistanceScannerRotZOnlySensor();
00034 
00035       virtual ~CFootBotDistanceScannerRotZOnlySensor() {}
00036 
00037       virtual void SetRobot(CComposableEntity& c_entity);
00038 
00039       virtual void Init(TConfigurationNode& t_tree);
00040 
00041       virtual void Update();
00042 
00043       virtual void Reset();
00044 
00045    private:
00046 
00047       void UpdateNotRotating();
00048       void UpdateRotating();
00049 
00050       Real CalculateReadingForRay(const CRay3& c_ray,
00051                                   Real f_min_distance);
00052 
00053       void CalculateRaysNotRotating();
00054       void CalculateRaysRotating();
00055 
00056    private:
00057 
00059       CRandom::CRNG* m_pcRNG;
00060 
00062       bool m_bAddNoise;
00063 
00065       CRange<Real> m_cNoiseRange;
00066 
00067       CSpace& m_cSpace;
00068       CFootBotDistanceScannerEquippedEntity* m_pcDistScanEntity;
00069       CControllableEntity* m_pcControllableEntity;
00070       CEmbodiedEntity* m_pcEmbodiedEntity;
00071       CRadians m_cLastDistScanRotation;
00072 
00073       bool m_bShowRays;
00074 
00075       CRay3 m_cShortRangeRays0[6];
00076       CRay3 m_cShortRangeRays2[6];
00077       CRay3 m_cLongRangeRays1[6];
00078       CRay3 m_cLongRangeRays3[6];
00079 
00080       /* Internally used to speed up ray calculations */
00081       CVector3 m_cDirection;
00082       CVector3 m_cOriginRayStart;
00083       CVector3 m_cOriginRayEnd;
00084       CVector3 m_cRayStart;
00085       CVector3 m_cRayEnd;
00086    };
00087 
00088 }
00089 
00090 #endif
```

---

Generated on 10 Jul 2018 for ARGoS by 
 1.6.1
